# Co-expression of NMDA-receptor subunits NR1, NR2A, and NR2B in dysplastic neurons of teratomas in patients with paraneoplastic NMDA-receptor-encephalitis: a retrospective clinico-pathology study of 159 patients

**DOI:** 10.1186/s40478-020-00999-2

**Published:** 2020-08-08

**Authors:** Xin-Yue Jiang, Song Lei, Le Zhang, Xu Liu, Min-Tao Lin, Ingmar Blumcke, Yue-Shan Piao, Dong Zhou, Jin-Mei Li

**Affiliations:** 1 Department of Neurology, West China Hospital, Sichuan University, 37th Guoxuexiang Road, Chengdu, Sichuan Province China; 2 Department of Pathology, West China Hospital, Sichuan University, Chengdu, Sichuan Province China; 3 Department of Neuropathology, University Hospital Erlangen, Erlangen, Germany; 4 Department of Neuropathology, Xuanwu Hospital, Capital Medical University, Beijing, China

**Keywords:** Dysplastic neurons, NMDA receptor, Teratoma, Chemokines, Cytokines

## Abstract

**Objective:**

To comprehensively describe the pathological features of neurons in patients with ovarian teratomas and paraneoplastic anti-NMDAR encephalitis (anti-NMDARE), emphasizing on NMDA-receptor expression and infiltrating lymphocytes.

**Methods:**

A retrospective study was performed in a large series of 159 patients from the West China Hospital. We retrospectively identified 12 patients with paraneoplastic anti-NMDARE (11 case with ovarian teratomas and 1 case with mixed germ cell tumor), which were compared to 35 patients with teratomas and no encephalitis and to 147 patients with anti-NMDARE and no evidence for tumors. Patient history and outcome were reviewed from the clinical charts and compared between all three groups. Histopathological examination, including double-immunofluorescence of NMDAR subunits and IgG was performed in all teratoma tissues. Magnetic Luminex Assay Human Premixed Multi-Analyte Kit was performed to investigate cytokines profile of CSF.

**Results:**

Patients with paraneoplastic anti-NMDARE had a more severe clinical presentation, i.e. they required more mechanical ventilation and intensive care (*p* < 0.001). Though immunotherapy was initiated earlier in this group, repeated intravenous immunoglobulin administration (IVIG) was more common (*p* = 0.002) and with higher cerebrospinal fluid (CSF) antibody titres (*p* = 0.004). Following tumor resection, the outcome did not differ between groups. A peculiar population of floating-frog like dysplastic neurons were observed only in teratomas of patients with paraneoplastic anti-NMDARE, co-expressing NR1, NR2A, NR2B subunits and IgG. Also, CD20 positive B-cells were more common in anti-NMDARE teratomas. In CSF of paraneoplastic anti-NMDARE patients, TNF-α, IL-10 and GM-CSF concentrations were higher than in negative symptom control and VEGF-A and IL-1a were lower than in anti-NMDARE patients (0.25 < *p* < 0.05).

**Conclusions:**

Patients with teratomas and paraneoplastic anti-NMDARE revealed a cellular population of dysplastic neurons co-expressing NMDAR subunits, which were the potential source of autoantigens triggering anti-NMDARE. Some inflammatory cytokines may be involved in pathogenesis of paraneoplastic anti-NMDARE.

## Introduction

Anti–N-methyl-D-aspartate receptor encephalitis (anti-NMDARE) is a major type of autoimmune encephalitis with more than 1000 reported cases since the discovery of autoantibodies against the NR1 subunit of the NMDA receptor in 2007 [9, 24]. In 4106 cases of encephalitis of unidentified aetiology in China, 423 cases (10.3%) were positive for anti-NMDAR antibodies [12]. Anti-NMDARE is also considered a paraneoplastic syndrome. Tumors, especially ovarian teratomas, exist in 38–59% of cases with anti-NMDARE in western countries [8, 19]. In China, ovarian teratomas were observed in 8% of all patients and 26.9% of women with anti-NMDARE [6, 22]. It has been suggested that anti-NMDAR antibodies were directed against neuronal cell surface antigens of specific tumor cell populations, i.e. dysplastic neurons, triggering the onset of anti-NMDARE with severe neurological symptoms following CNS distribution via cerebrospinal fluid (CSF) [7]. Previous studies described also abundant lymphocyte infiltration in teratoma tissues [5, 13, 20], suggesting that an inflammatory response is caused by these teratomas. Though brain tissue is difficult to obtain from such patients, histopathological specimens from resected teratomas are a valuable source for analysis in order to better understand disease pathomechanism. Our current study aimed to explore the relationship between teratomas and anti-NMDARE by systematically reviewing pathology, clinical features, and inflammatory cytokines.

## Materials and methods

### Patients

Between February 2013 and October 2017, 3020 patients were admitted with clinical suspicion of autoimmune encephalitis to the West China Hospital in Chengdu. Serum and CSF samples were taken from each patient and sent to Peking Union Medical College Hospital (China) for screening of autoimmune-antibodies, including antibodies to NMDAR, contactin-associated protein-like 2 (CASP2), leucine-rich glioma inactivated 1 (LGI1), gamma aminobutyric acid beta receptor (GABAB-R), glutamic acid decarboxylase (GAD65), alpha-amino-3-hydroxy-5-methyl-4-isoxazole -propionic acid 1/2 receptor (AMPA1/2-R), IgLON5, dipeptidyl-peptidase-like protein-6 (DPPX), metabotropic glutamate receptors (mGLURs), voltage gated potassium channel (VGKC), and voltage gated calcium channel (VGCC). All serum and CSF specimens were evaluated by indirect immunofluorescence using EU 90 cells transfected with the NMDAR1 subunit (NR1) of the NMDAR complex and immobilized on BIOCHIPs (Euroimmun AG, L €ubeck, Germany) as previously described [17]. Finally, a diagnosis of anti-NMDARE was confirmed in 159 patients [11]. Antibody titres in CSF and serum were classified as weakly positive (CSF 1:1, serum 1:10), positive (CSF 1:10 or 1:32, serum 1:32), and strongly positive (CSF 1:100 or 1:320, serum 1:100). Twelve of the patients with anti-NMDARE, who suffered from a teratoma and received operation, were included into the study (the paraneoplastic anti-NMDARE group). In addition, we identified 64 age-matched patients (age range 17 to 43 years) without neurological dysfunction who had teratomas resected between Jun 1st and Jun 30th in 2018 in the Department of Gynecology, West China Second Hospital, Sichuan University. Histopathologically, a total of 35 teratomas revealed central nervous tissues and these samples were selected as a control group. In addition, patients with anti-NMDAE were included as the anti-NMDAE controls (N) and patients who diagnosed as chronic headache with normal MRI and CSF test were included as the negative symptom controls (C) for CSF cytokines and chemokines detection.

### Clinical data review

Details of the demographics, clinical manifestations, results of auxiliary examinations, treatment strategies, and outcomes were reviewed from the hospital archives. A modified Rankin Scale (mRS) was used to evaluate the general outcome at the final follow-up. A good outcome was defined as mRS score 0–2 [21]. Relapse of encephalitis was defined as new onset or worsening of symptoms occurring after an initial improvement or stabilization of at least 2 months [19].

### Sample preparation

After CSF were obtained during the acute phase of encephalitis, all samples were centrifuged at 1000 r/min for 10 min. Subsequently, the supernatants were stored at − 20 °C for up to 1 month and then in liquid nitrogen until further use.

Formalin-fixed and paraffin-embedded blocks of tumor tissue were obtained from the Department of Pathology, West China Hospital/ West China Second Hospital. Four μm thin sections were cut with a microtome and used for further analysis (see below).

### Pathological examination

For every tumor block, five sections were cut and reviewed by Hematoxylin-Eosin (HE) staining and immunohistochemistry of Neuronal Nuclear epitope (NeuN), Microtubule Associated Protein 2 (MAP2), S100 (S100), and Glial Fibrillary Acidic Protein (GFAP). The tumor blocks with confirmed central nervous system tissues were prepared for next step of experiment. All sections were reviewed using Hematoxylin-Eosin (HE) staining and further processed for immunohistochemistry and immunofluorescence double-staining using standardized protocols. Lymphocytes were detected with antibodies directed against CD20 specific for B-cells, CD138 for plasma cells and against CD3, CD4 and CD8 for T-cells. Antibodies directed against immunoglobulin G (IgG), NR1, NR2A, and NR2B were used as specified in Supplementary Table 1. Thirty sections were cut from every tumor and six sections from the 1st, 7th, 13th, 19th, 25th and 30th section was chosen for NR1/NR2A/NR2B immunofluorescence. To identify the cellular source of IgG binding, immunofluorescence double-staining was performed with NR1/NR2A/NR2B receptors. FITC (fluorescein isothiocyanate) and TMRITC (trimethyl rhodamine isothiocyanate) were used to label IgG and NR1/NR2A/NR2B receptors, respectively, and visualized with a fluorescence microscope (Zeiss, AX10 imager A2/AX10 cam HRC). We observed the whole field and screened five surface per section fluorescence of co-localization after merging. Yellow co-localization cells were evaluated under 400 multiple.

The immunohistochemical and double immunofluorescence results were independently evaluated by one pathologist and two neurologists who were blinded to all experimental groups.

### Chemokine and cytokine detection in CSF

We applied the Magnetic Luminex Assay Human Premixed Multi-Analyte Kit (R&D Systems, Minneapolis, MN, USA) to measure the concentration of the following chemokines and cytokines (according to the manufacturer’s instructions): chemokines C-C motif chemokine ligand 2 (CCL2), C-X-C motif chemokine ligand 1 (CXCL1), CXCL9, CXCL10 and CXCL13, and the cytokines interferon (IFN)-α, IFN-γ, interleukin (IL)-1α, IL-1β, IL-2, IL-4, IL-5, IL-6, IL-8, IL-10, IL-12, IL-17A, tumour necrosis factor (TNF)-α, vascular endothelial growth factor A (VEGF-A), a proliferation-inducing ligand (APRIL), B-cell-activating factor belonging to the tumour necrosis factor family (BAFF), granulocyte colony-stimulating factor (G-CSF), and granulocyte-macrophage colony-stimulating factor (GM-CSF). All data were collected with the Luminex-100 system (Luminex, Austin, TX, USA).

### Statistical analyses

Statistic Package for Social Science (SPSS) version 22.0 (SPSS Inc. Chicago, IL, USA) was used for statistical analyses. The cytokine/chemokine levels of paraneoplastic anti-NMDARE patients were compared with anti-NMDARE patients and negative symptom controls respectively by the Mann-Whitney U test and Bonferroni correction (*P* < 0.025 was considered as significant). Mann-Whitney U test was also applied for other continuous variables that were not normally distributed and the chi-square test or Fisher’s exact test for categorical variables. Differences with a *p*-value < 0.05 were considered as significant.

## Results

### Clinical features and demographics

A total of 159 patients were included in this study and divided into paraneoplastic anti-NMDARE and anti-NMDARE groups. The study group (12 cases) included 11 women and 1 man, and the median age was 25 years (range: 17–43). In these patients, the first five common symptoms were seizures (12/12), cognitive dysfunction (12/12), psychiatric behaviors (11/12), decreased levels of consciousness (10/12), and central hypoventilation (8/12). The median length of hospital stay of these patients was 57 days (range: 15–385). Electroencephalography (EEG) showed slow waves and/or epileptiform discharges as well as extreme delta brushes in 11 patients. Neuroimaging results showed abnormal signaling in T2 or fluid-attenuated inversion recovery (FLAIR) in the cortex or the white matter, meningeal enhancement, and ventricle enlargement in 5 patients. Routine CSF tests showed elevated pressure (> 180 mm H_2_O) and a slightly elevated white blood cell count (> 5/μl) and/or increased proteins (> 500 mg/L) in six patients. Immunotherapy including IVIG, methylprednisolone and rituximab were used in addition to tumor resection in all patients. Two cases received chemotherapy (VBC: vincristine, bleomycin, and cis-platinum) after the histopathology diagnosis revealed an immature teratoma. The median follow-up duration was 26 months (range 1–66 months). Eleven patients (91.7%) had a good outcome (mRS 0–2) and 1 patient died. This patient (case 12) suffered from fast tumor growth and pulmonary metastases, and died of respiratory failure in the third month after onset. All demographic and clinical data were summarized in Table 1.

**Table 1:** Clinical data of 12 patients with paraneoplastic anti-NMDA receptor encephalitis

NO	Sex/ age	Hospital stay/d	Prodromal symptom	Psychiatric symptoms	Seizure	Cognitive dysfunction	Speech dysfunction	Movement disorder	Consciousness decreased	Autonomic dysfunction	Central hypoventilation	MRI	EEG	CSF	Anti-NMDAR antibody		Immunotherapy	mRs of follow-up
															CSF	Serum		
N-1	f/18	57	Headache	Early irritability, manic, aggression and automutilation, disorganized, Late Psychomotor inhibition	GTCS SE	Yes	pressured speech and mutism	choreoathetoid movements	coma	hypotension	Yes	Normal	focal slow activity	Increased white blood cells	+++	++	IVIG 5d * 2	0 (2.5 Year)
N-2	f/19	106	Fever	hallucination, agitation, irritability	GTCS	Yes	pressured speech	Orofacial dyskinesia, Myoclonus	delirium, light coma	pyrexia	Yes	Bilateral temporal lobe meningeal thickening	diffuse slow activity and epileptic activity	Increased white blood cells	+++	–	IVIG 5d * 2 Methylprednisolone	0 (4.2 Year)
N-3	f/17	50	Fever	Disorganized, agitation, manic, insomnia, hallucinations	GTCS	Yes	No	Orofacial dyskinesia	coma	No	No	Normal	epileptic activity	Increased white blood cells	++	+++	IVIG 5d * 2 Methylprednisolone 1000 mg 5d	0 (2.5 Year)
N-4	f/17	73	Have a cold	Agitation, Hallucinations, Delusions	GTCS	Yes	No	No	coma	pyrexia, tachycardia	Yes	meningeal enhancement	focal slow activity	0	+++	+++	Methylprednisolone 500 mg 2d IVIG 20 g 5d*2	0 (5.5 Year)
N-5	f/28	70	No	Agitation, Hallucinations, Delusions, automutilation	GTCS	Yes	gibberish-speaking	Orofacial dyskinesia, choreoathetoid movements	somnolence, deliration	No	Yes	Normal	diffuse slow activity and extreme delta brush	Increased white blood cells	+++	++	IVIG 5d * 3 Methylprednisolone 1000 mg 3d	1 (1.5 Year)
N-6	f/20	28	Headache	Disorganized, agitation	CPS	Yes	No	Orofacial dyskinesia	light coma	tachycardia	Yes	–	diffuse slow activity and extreme delta brush	Increased white blood cells and Protein	+++	_	IVIG 5d * 3 Methylprednisolone 1000 mg 5d	0 (2.4 Year)
N-7	f/22	27	insomnia	Agitation, Hallucinations, Delusions, Disorganized, automutilation	CPS	Yes	No	Orofacial dyskinesia	No	urinary incontinence	No	Normal	diffuse slow activity	0	+++	++	IVIG 5d Methylprednisolone 500 mg 5d	0 (1.6 Year)
N-8	f/20	58	numbness of fingers	No	SGTCS	Yes	No	finger spasm	No	No	No	Normal	focal slow activity	Increased white blood cells	+++	++	IVIG 5d*2	1 (2 Year)
N-9	f/29	400	Fever	Hallucinations	GTCS	Yes	–	Orofacial dyskinesia	coma	No	Yes	Abnormal signals in bilateral frontal and temporal lobe	focal slow activity and epileptic activity	Elevated pressure and increased white blood cells	+++	++	IVIG 5d * 4 Rituximab 500 mg * 3 Methylprednisolone 1000 mg 5d	1 (1 Year)
N-10	f/35	23	No	Aggression, Disorganized, Agitation, Manic, Insomnia, Hallucinations, Psychomotor inhibition	CPS SE	Yes	mutism	No	coma	No	Yes	cerebral infarction in Splenium of Corpus Callosum, Right lateral ventricle enlargement	extreme delta brush	0	+++	++	IVIG 5d	0 (3.6 Year)
N-11	f/43	15	No	Aggression, Disorganized, Agitation, Manic	GTCS	Yes	No	No	somnolence	No	No	Normal	diffuse slow activity and extreme delta brush	0	+++	_	IVIG 5d	0 (2 Year)
N-12	m/25	113	Have a cold	Disorganized, Agitation Manic	GTCS	Yes	disfluency	Orofacial dyskinesia	coma	pyrexia	Yes	Abnormal signals in bilateral temporal lobe and amygdala	Normal	Elevated pressure and increased white blood cells	+++	+	IVIG 5d*2	6

*f* female, *m* male, *GTCS* generalized tonic-clonic seizure, *SE* status epilepticus, *CPS* complex partial seizure, *SGTCS* secondary generalized tonic-clonic seizure, negative (−), weakly positive (+, CSF 1:1, serum 1:10), positive (++, CSF 1:10 or 1:32, serum 1:32), strongly positive (+++, CSF 1:100 or 1:320, serum 1:100)

Comparisons of clinical features between the paraneoplastic anti-NMDARE and anti-NMDARE groups showed that teratomas occurred more often in women than men (91.7% vs 53.1%; *p* = 0.01). The paraneoplastic anti-NMDARE group received more often central hypoventilation compared to the anti-NMDARE group (66.7% vs 17.0%; *p* < 0.001), required more intensive care (58.3% vs 8.8%, *p* < 0.001) and the length of their hospital stay was longer (57.5 days vs 24 days; *p* = 0.001). The paraneoplastic anti-NMDARE group also had higher CSF antibody titres (*p* = 0.004). Though the paraneoplastic anti-NMDARE group received immunotherapy earlier (interval from onset to immunotherapy: 12 days vs 25 days, *p* = 0.002), repeated immunotherapy was more commonly used than in the control group (2 times IVIG vs 1 time IVIG, *p* = 0.002). However, after tumor resection, the final outcome and relapse rate did not differ between the two groups. Comparisons of clinical features between the paraneoplastic anti-NMDARE and anti-NMDARE groups were summarized in Table 2.

**Table 2:** Comparisons between paraneoplastic anti-NMDARE and anti-NMDARE groups without teratoma

	Total, n(%)	Paraneoplastic anti-NMDARE, n(%)	Anti-NMDARE without teratoma, n(%)	*P* values
Total	159	12 (7.5%)	147 (92.5%)	–
Age, years, median (range)	25(9–78)	21 (17–43)	26 (9–78)	0.445^b^
Sex (female)	89(56.0%)	11 (91.7%)	78 (53.1%)	0.01^a^
Psychiatric symptoms	151 (95%)	11 (91.7%)	140 (95.2%)	0.474^c^
Seizure	130 (81.8%)	12 (100%)	118 (80.3%)	0.125^c^
Speech disturbances	60/141(42.6%)	5/11 (45.6%)	55/130 (42.3%)	1^c^
Dyskinesias and movement disorders	90 (56.6%)	9 (75%)	81 (55.1%)	0.384^a^
Autonomic instability	53 (33.3%)	6 (50%)	47 (32.0%)	0.53^c^
Memory deficits	97/119 (81.5%)	6/8 (75.0%)	91/111 (82.0%)	0.64^c^
Decreased consciousness	97 (61.0%)	10 (83.3%)	87 (59.2%)	0.129^c^
Central hypoventilation	33 (20.8%)	8 (66.7%)	25 (17.0%)	< 0.001^c^
Complications	106 (66.7%)	10 (83.3%)	96 (65.3%)	0.34^c^
Abnormal MRI findings	61/148 (41.2%)	5/11 (45.5%)	56/137 (40.9%)	0.761^c^
Abnormal EEG findings	113/137 (82.5%)	11/12 (91.6%)	102/125 (81.6%)	1^c^
Abnormal CSF findings	102 (64.2%)	8 (66.7%)	94 (63.9%)	1^c^
Antibody titre in CSF				
Weakly positive	8	0	8	0.004 ^b^
Positive	70	1	69	
Strongly positive	81	11	70	
Antibody titres in serum				
Negative	69/157	3	66/145	0.114 ^b^
Weakly positive	17/157	1	16/145	
Positive	58/157	6	52/145	
Strongly positive	13/157	2	11/145	
Treatment				
IVIG(times)	1 (0–4)	2 (1–4)	1 (0–3)	0.002 ^b^
MTP(times)	1 (0–3)	1 (0–2)	1 (0–3)	0.991 ^b^
IVIG+MTP(times)	2 (1–5)	3 (1–5)	2 (1–5)	0.037 ^b^
IVIG+MTP + RTX(times)	2 (1–8)	3 (1–8)	2 (1–8)	0.042 ^b^
Interval from onset to receive IT, days, median (IQR)	24 (4–365)	12c (7–75)	25 (4–365)	0.002 ^b^
In hospital days, median (IQR)	25 (3–400)	57.5 (15–400)	24 (3–118)	0.001 ^b^
Need of ICU	20(12.6%)	7(58.3%)	13 (8.8%)	< 0.001^c^
Outcome				
Good outcome (mRS = 0–2)	125/144 (86.8%)	11/12 (91.7%)	114/132 (86.4%)	1^c^
Death	12/144 (8.3%)	1/12 (8.3%)	11/132 (8.3%)	1^c^
Relapse	13/144 (9.0%)	1/12 (8.3%)	12/132 (9.1%)	1^c^

*CSF* cerebrospinal fluid, *IVIG* intravenous immunoglobulin, *MTP* methylprednisolone, *RTX* rituximab, *IQR* interquartile range, *ICU* intensive care unit, *mRS* modified Rankin Scale

^a^Chi-squared test

^b^Mann-Whitney U test

^c^Fisher’s exact test

### Histopathology findings

The 12 patients with anti-NMDARE presented with the following spectrum of histopathology diagnosis: mature teratomas of the ovary (case 2–9), mature teratoma of the mediastinum (case 1), immature teratomas of the ovary (WHO III, cases 10–11), and a mixed germ cell tumor of the mediastinum (case 12). The size of the teratomas ranged from 2.1 × 2 × 1.9 cm to 18.5 × 10.3 × 9 cm in patients with anti-NMDARE (Table 3) and from 2 × 1.8 × 1 cm to 16.5 × 13 × 8.5 cm for the control group (Supplementary Table 2). HE staining revealed a characteristic spectrum of mature elements including skin, gastrointestinal tissue, muscle, cartilage, and sebaceous tissue accompanied by mature or immature neural elements.

**Table 3:** Surgery finding and inflammatory features of teratomas in patients with anti-NMDARE

Group	Age	Sex	Pathology findings	Location	IgG	CD20	CD138	CD3	CD4	CD8
N1	19	f	2.2 × 4.9 × 5.7 cm Cystic mature teratoma	Left ovary	+	++	+	+	–	–
N2	18	f	4.7 × 5 × 6.8cmCystic mature teratoma	thoracic cavity	+++	+++	–	+++	++	+
N3	17	f	3 × 3 × 2 cm Cystic mature teratoma	Left ovary	++	++	–	+	+	–
N4	17	f	Cystic mature teratoma	Right ovary	++	+	–	++	++	++
N5	28	f	2.1 × 2 × 1.9 cm Cystic mature teratoma	Right ovary	+++	–	–	+	–	–
N6	20	f	3.7 × 3 × 2.9 cm Cystic mature teratoma	Left ovary	+++	+++	–	+	–	–
N7	22	f	1.5 × 1.2 × 1.6 cm Cystic mature teratoma	Left ovary	++	++	–	++	–	–
N8	20	f	4 × 3 × 1.1 cm Cystic mature teratoma	Left ovary	++	++	–	+	++	+
N9	29	f	Cystic mature teratoma	Left ovary	+++	+	–	–	–	+
N10	35	f	6.8 × 5.4 × 4.5 cm Immature teratoma (WHO III)	Left ovary	++	++	+	+	–	–
N11	43	f	18.5 × 10.3 × 9 cm Immature teratoma (WHO III)	Left ovary	+++	+++	–	+	–	+
N12	25	m	6.7 × 5 × 5.4 cm Mixed germ cell tumor (choriocarcinoma with teratoma)	Mediastinum	+++	+++	++	+	+++	+

N, patients with paraneoplastic anti-NMDARE; *f* female, *m* male, *IgG* immunoglobulin, Cellular infiltrates: -, negative or positive cells less than 1% of microscopic field; +, < 25%; ++, 25–50%; +++, 50–75%; ++++, > 75%. positive IgG deposit: -, absent; +, mild; ++, moderate; +++ intense

**Fig. 1:**
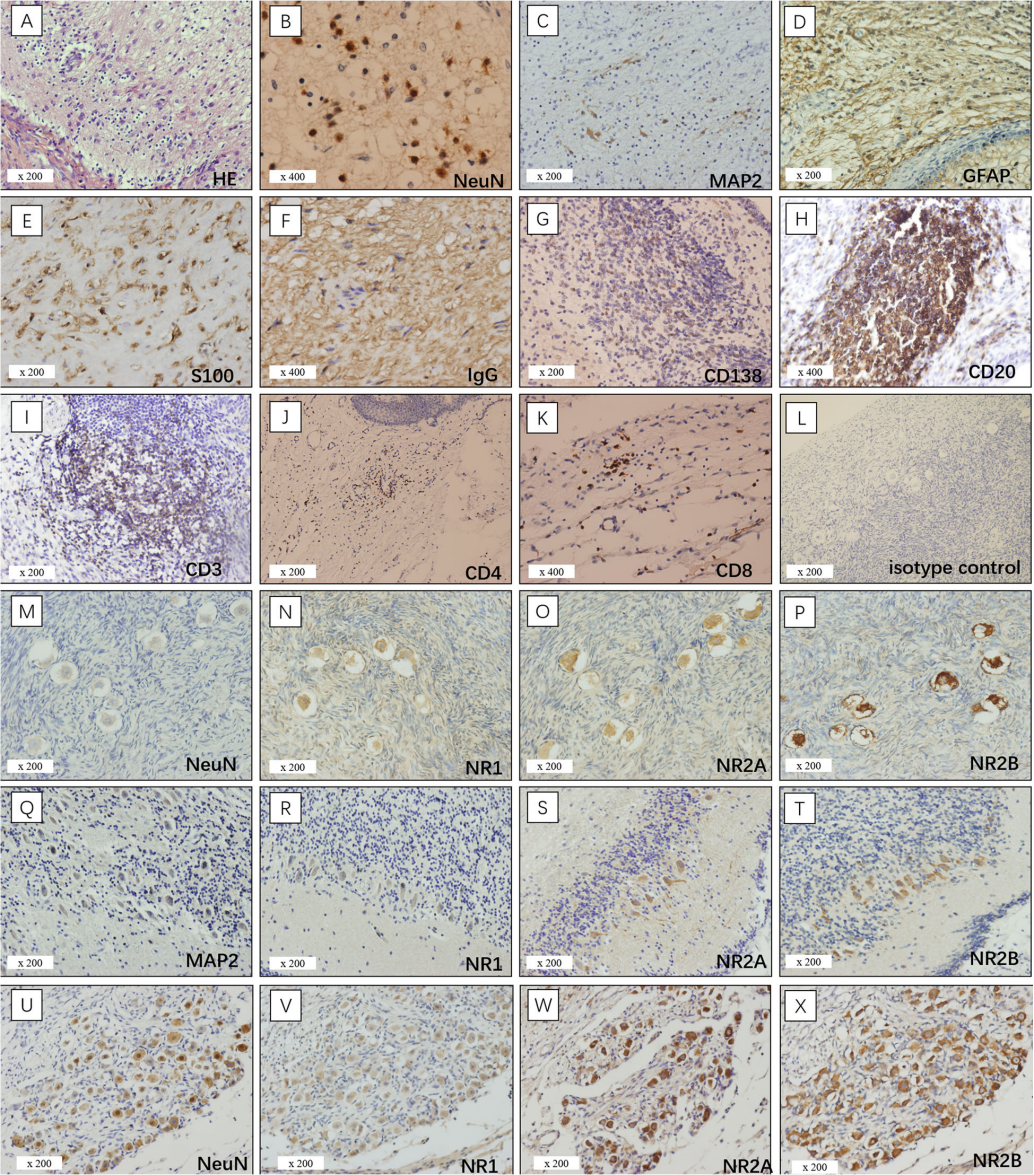
Pathology findings of neural tissues and NMDAR staining in teratomas with anti-NMDARE. Pathology findings of neural tissues in teratomas and NMDAR staining from patients with anti-NMDARE. (**a**) HE staining showed degenerative neurons. (**b**) Normal neurons with enlarged nuclei. (**c**) Neural cells positive for MAP2. (**d**) Neuroglia positive for GFAP. (**e**) Neural cells positive for S100. (**f**) In case N2, IgG positivity is strong and abundant. (**g**) Plasma cells positive for CD138. (**h**) and (**i**) Nodules of lymphocytes positive for CD20 and less positive for CD3, respectively. (**j**) Less helper T lymphocytes positive for CD4. (**k**) Cytotoxic T lymphocytes positive for CD8. (**l**), Isotype control of case N6, (**m**) Mimicking floating-frog dysmorphic neurons showing positivity for NeuN. (**n**-**p**) Moderate NR1/NR2A positivity and strong NR2B positivity in serial sections to M. (**q**) In case N7, scattered dysmorphic neurons positive for MAP2. (**r**-**t**) Moderate NR1 positivity and strong NR2A/NR2B positivity in serial sections to Q. (**u**) In case N10,clusters dysmorphic neurons positive for NeuN. (**v**-**x**) Moderate NR1 positivity and strong NR2A/NR2B positivity in serial sections to U

NeuN staining of neural tissue in the tumor study group showed following patterns (Table 4): (I) neural tissues with normal mature neurons; (II) dysmorphic neurons with floating-frog neural elements; (III) clusters of dysmorphic neurons; (IV) scattered dysplastic neurons; (V) small cells with enlarged nuclei positive for NeuN in 1 case (N12). The dysmorphic neurons showed an irregular cell shape with giant nuclei. Eleven of the 12 cases (91.7%) also tested positive for MAP2 and S100. Pathological findings of neural tissues in teratomas from patients with NMDARE and their staining for NR1, NR2A and NR2B were shown in Fig. 1.

**Table 4:** NMDAR subunit analysis in neurons of teratomas with/without anti-NMDARE

Neuron morphology	Case	Neuron staining	NR1	NR2A	NR2B
Normal neurons	N1	NeuN+	+	++	+++
	N2	NeuN+	+	++	+
	N7	NeuN+	–	–	–
	N8	NeuN+	++	–	+++
	N10	NeuN+	–	–	–
	N11	NeuN+	+	+	+++
Mimicking floating-frog dysmorphic neurons	N4	NeuN+	+	++	+++
	N5–6	NeuN+	++	+++	+++
	N9	S100++ MAP2+	+	–	+++
Clusters of dysmorphic neurons	N2	NeuN+	+	+++	+++
	N3	NeuN+	–	+	++
	N7	NeuN+	++	++	++
	N10	NeuN+	+	+++	+++
Scattered dysmorphic neurons;	N1	NeuN+	+	++	+++
	N7	MAP2+	+	+++	++
	N8	NeuN+	++	+	+++

N1–11: patients with paraneoplastic anti-NMDARE; C1–12: patients without anti-NMDARE; −, positive cells less than 1% of microscopic field; + < 25%; ++, 25–50%; +++, 50–75%; ++++, > 75%. *NeuN* Neuronal Nuclear epitope, *MAP2* Microtubule Associated Protein 2, *GFAP* Glial Fibrillary Acidic Protein, *NR* NMDA reception

Inflammatory cell infiltrates were composed of CD20- and CD3-positive lymphocytes. The lymphocytes usually clustered in nodules with intensive staining for CD20-positive B cells at the center, and CD3 positive T-cells at the periphery. In teratomas with anti-NMDARE, inflammatory cells with a CD20-positive lymphocytic wall adjacent to the neuropil were seen in 1 case. CD20-positive lymphocytic walls of 2–3 layers around the vasculature were found in 2 cases. More CD20-positive B-cells and less CD4-positive helper T-cells were present in the teratomas associated with anti-NMDARE than in the controls (CD20: *p* = 0.001, CD4: *p* = 0.009, Table 3 and Supplemental Table 2), but there was no statistical difference regarding CD138-positive plasma cells, CD3-positive T-cells and CD8-positive cytotoxic T-cells between the two groups (CD138: *p* = 0.521; CD3: *p* = 0.293; CD8: *p* = 0.386).

**Fig. 2:**
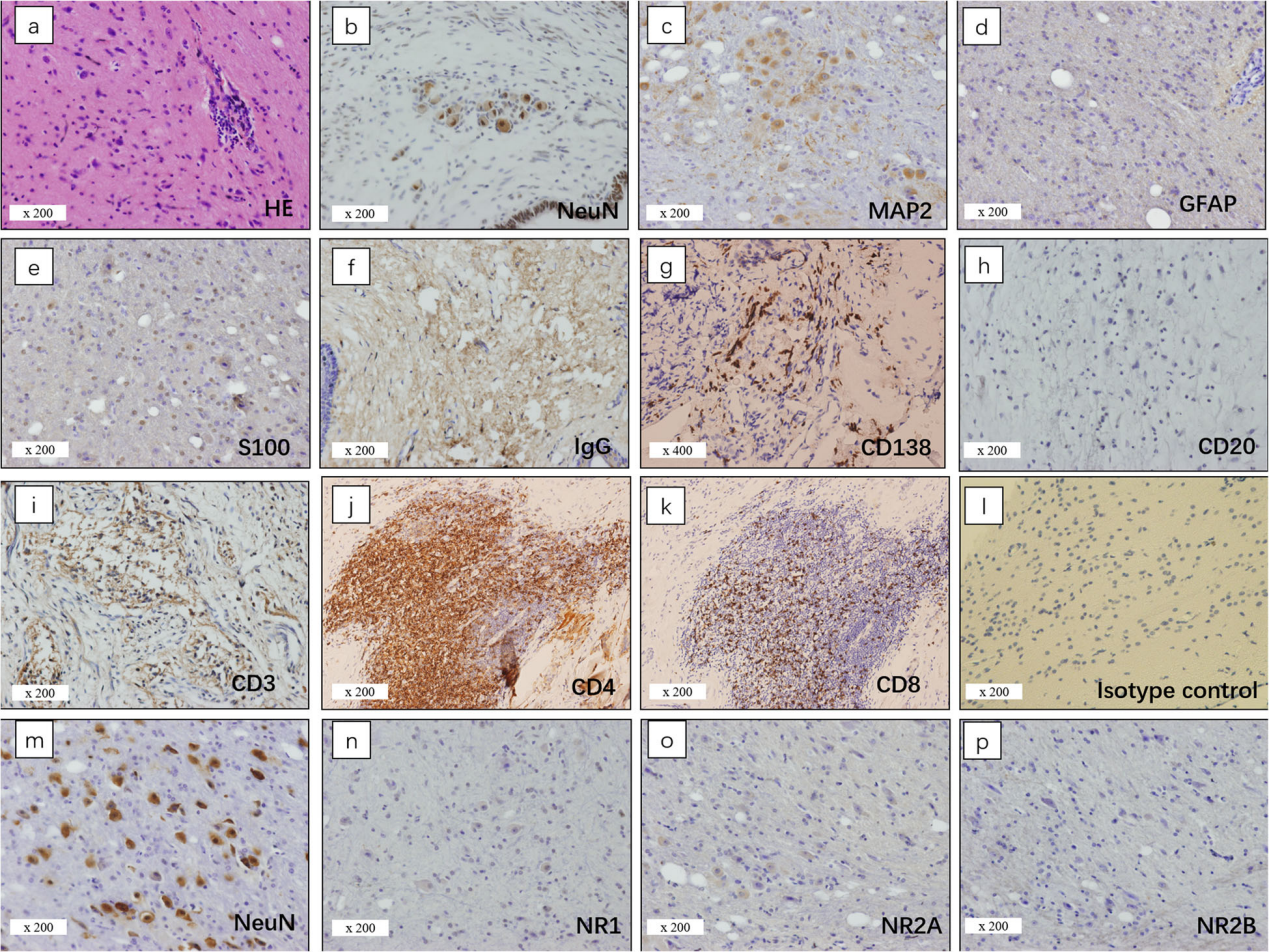
Pathology findings of neural tissues and NMDAR staining in teratomas without anti-NMDARE. Pathology findings of neural tissues in teratomas and NMDAR staining from patients without anti-NMDARE. (**a**) HE staining showed neurons with neuropil. (**b**) a pitch of dysmorphic neurons with enlarged nuclei. (**c**) Aggregates of neurons positive for MAP2. (**d**) Neuroglia with moderate positivity for GFAP. (**e**) Scattered cells with moderate positivity for S100. (**f**) IgG positivity is moderate and spreading. (**g**) Plasma cells positive for CD138. (**h**) and (**i**) Less positive for CD20 and clusters of lymphocytes positive for CD3. (**j**) Helper T lymphocytes positive for CD4. (**k**) Cytotoxic T lymphocytes positive for CD8. (**l**) isotype control of case C2. (**m**) scattered dysmorphic neurons positive for NeuN. (**n**–**p**) no immunostaining for NR1, NR2A, and NR2B in serial sections of the control case

**Fig. 3:**
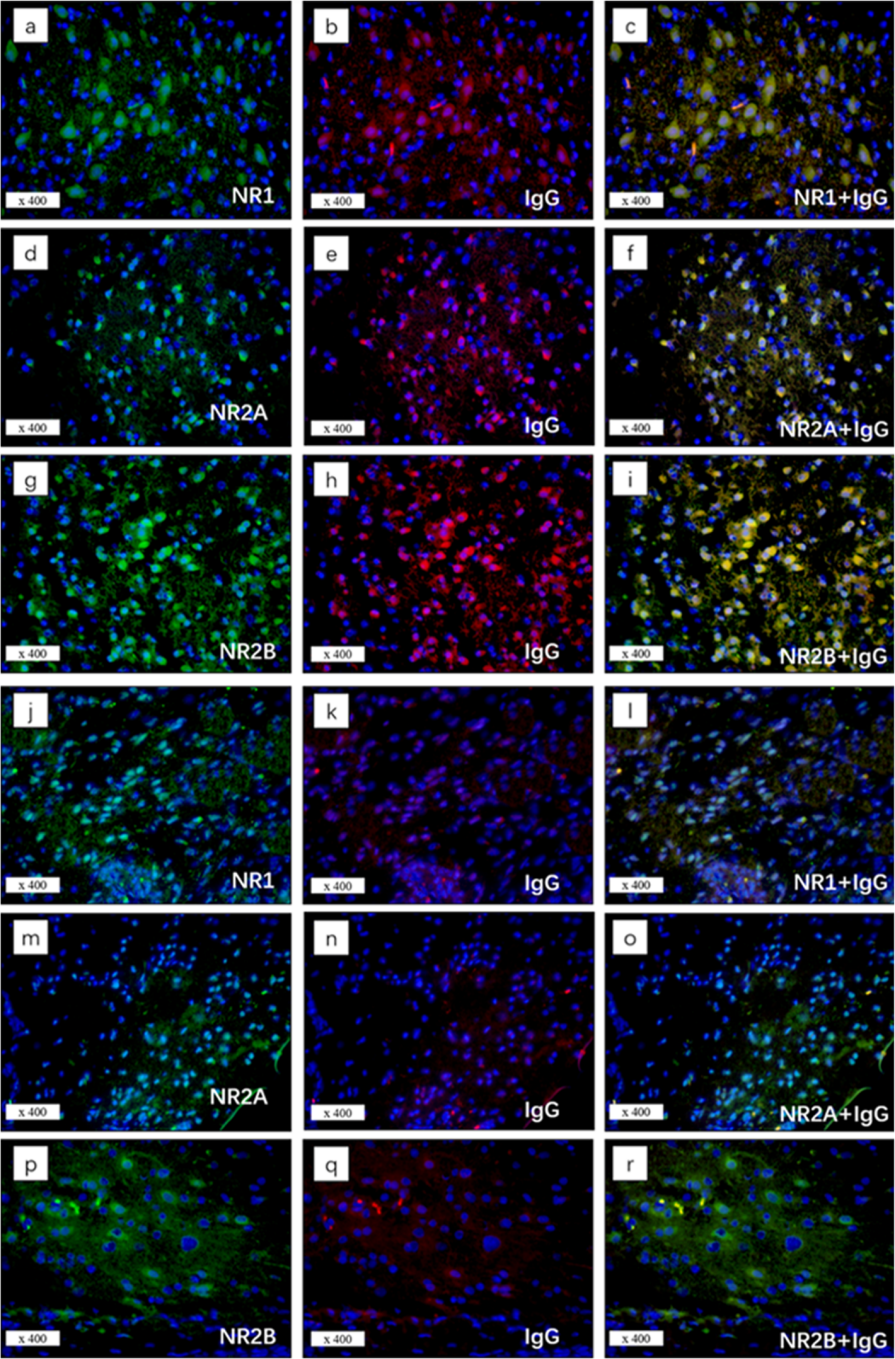
Immunofluorescence staining revealed co-localization of NR1/NR2A/NR2B with IgG in neural tissue of teratomas. Immunofluorescence staining revealed co-localization of NR1/NR2A/NR2B (green) with IgG (red) in neural tissue of teratomas with anti-NMDARE (**a**-**i**) and without anti-NMDARE (**j**-**r**). (**a**, **d**, **g**) Green fluorescence staining for NR1, NR2A, andNR2B, respectively. (**b**, **e**, **h**) IgG staining with red fluorescence. (**c**. **f**. **i**) co-localization of IgG with NR1, NR2A, and NR2B. (**j**-**r**) no co-localization of IgG with NR1/NR2A/NR2B in neural tissue of teratomas without anti-NMDARE

In the control group, all cases showed neural tissues consisting of mature neurons. One cases (C12) had an additional small pitch of dysplastic neurons. Mature neurons in twenty cases stained positive for NMDAR subunits expression (NR1: *n* = 9; NR2A: *n* = 16; NR2B: *n* = 11) while fifteen cases were negative for NR1, NR2A or NR2B immunoreactivity. Interestingly, dysplastic neurons in C12 did not show any NR1, NR2A or NR2B positivity. GFAP expression was positive in all patients from the study and control groups. Figure 2 summarized the pathological features of the control group. Ten of 12 samples of patients with paraneoplastic anti-NMDARE showed moderate or intense expression of NR1 and NR2A /NR2B subunits, while N3 was positive only for NR2A and NR2B and N9 was positive only for NR1 and NR2B. NR1, NR2A and NR2B expression was stronger in the anti-NMDARE group than in the control group (NR1: *p* < 0.001; NR2A: *p* < 0.001; NR2B: *p* < 0.001, Table 4 and Supplemental Table 3). There was no significant difference between the two groups regarding the expression of IgG (*p* = 0.069, Table 3 and Supplemental Table 2). In the encephalitis associated teratomas with dysmorphic neurons, all NMDAR subunits and IgG were detected in the same visual field. Subsequent immunofluorescence staining showed IgG reactivity substantially co-localized with the reactivity of NR1, NR2A, and NR2B antibodies in patients with paraneoplastic anti-NMDARE. In the control group, no sample exhibited NR1, NR2A and NR2B reactivity with IgG (Fig. 3).

**Fig. 4:**
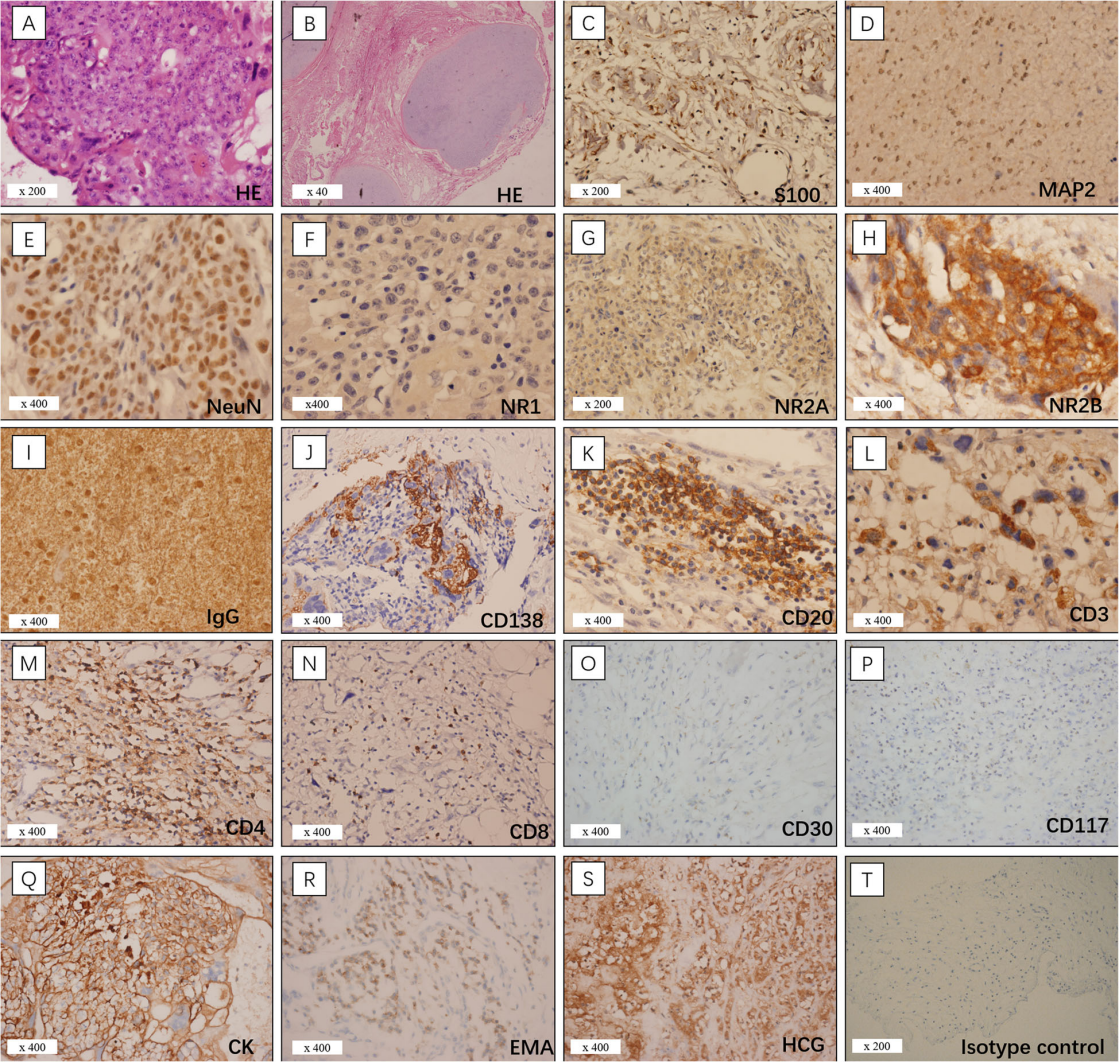
The pathology findings of the patient with a mixed germ cell tumor (case N12). (**a**) Increased tumor cell density with abundant mitoses. (**b**) HE staining showing the teratoma element with cartilage. (**c**–**d**) MAP2 and S100 positive cells were scattered. (**e**) Atypically composed neurons of high density and irregular nuclei. (**f**–**g**) Negative staining for NR1 and moderate positive staining for NR2A. (**h**) Strongly positive staining for NR2B in cells clustered as a nest. (**i**) Strong staining for IgG. (**j**) Plasma cells positive for CD138. (**k**) Clustered lymphocytes positive for CD20. (**l**) T-lymphocytes positive for CD3. (**m**) Helper T lymphocytes positive for CD4. (**n**) Cytotoxic T lymphocytes positive for CD8. (**o**-**p**) Negative staining for CD30 and CD117, which used as marker of embryonal carcinoma and seminoma, dysgerminoma, germinoma. (**q**-**r**) Positive staining for CK and negative staining for EMA. (**s**) Tumor shows strong positive staining for HCG. (**t**) Isotype control

Case N12 was diagnosed with a mixed germ cell tumor (choriocarcinoma with teratoma) and the patient died of multiple organ failure within 3 months. The sections from this patient showed very different pathological characteristics to other patients (Fig. 4). On HE sections, tumor cells formed an intensive cluster with increased mitosis. NeuN-positive cells had an atypically closed neuronal shape with high density and irregular nuclei. MAP2- and S100-positive cell staining was diffuse. Lymphocytes clustered as foci with numerous intensive CD20 and CD138-positive cells, while intensive CD3-positive cells were separately distributed around neurons. Moderate CD4, CD8-positive, NR2A-positive cells, strong staining of diffusing IgG and clustered NR2B-positive cells were observed. Cell necrosis was also obvious in this sample. Other immunolabelled results included PLAP(+, focal), OCT3/4(−), glypican-3(−), CD117(−), CD30(−), AFP(−), HCG(+), HPL(+, focal), CK(+), and EMA(−) (Fig. 4).

### Chemokines and cytokines in CSF

As shown in Table 5, we detected higher levels of TNF-α, IL-10 and GM-CSF in CSF of paraneoplastic anti-NMDARE patients compared to the negative symptom control group (*P* < 0.05)and lower level of IL-1α and VEGF-A compared to the anti-NMDAR encephalitis patients(*P* < 0.05). However, there was no statistically significant difference after Bonferroni correction as the comparison were performed twice.

**Table 5:** Cytokines/Chemokines in CSF of patients with paraneoplastic anti-NMDAR encephalitis and anti-NMDAR encephalitis without teratoma, negative symptom controls

Cytokines/ Chemokines	Paraneoplastic anti-NMDARE (P, medium, range; pg/ml)	Anti-NMDARE without teratoma (N, medium, range; pg/ml)	Negative symptom control (C, medium, range; pg/ml)	*P* value	
				P vs N^a^	P vs C^a^
TNF-α	3.19(2.83–3.55)	3.19(2.32–8.15)	2.49(1.84–3.55)	0.613	0.039
IFN-γ	47.68(46.03–59.35)	46.03(30.12–59.35)	42.77(37.94–57.67)	0.286	0.083
CXCL9	174(121–336)	227(121–443)	227(121–336)	0.246	0.724
CXCL10	69.03(3.53–147)	15.6(1.82–7002)	4.49(2.26–147)	0.793	0.088
IL-2	415(345–458)	460(318–630)	408(327–564)	0.324	0.831
IL-4	16.17(11.77–31.30)	26.07(11.77–49.06)	17.72(8.66–36.3)	0.099	0.914
IL-5	1.83(1.68–2.25)	1.97(1.13–4.5)	1.68(1.41–2.25)	0.732	0.424
GM-CSF	12.56(4.43–18.44)	5.31(2.77–69.48)	4(2.02–18.44)	0.356	0.025
G-CSF	26.07(15.37–30.44)	26.9(15.37–110)	27.62(18.59–41.01)	0.429	0.52
IL-6	1.33(0.88–11.33)	3.26(1.15–68.3)	1.73(1.15–11.33)	0.158	0.392
IL-8	50.79(17.89–87.97)	58.47(11.28–150)	25.54(3.96–87.97)	0.694	0.055
IL-17A	0.81(−0.81)	0.81(−5.02)	0.81(−2.79)	0.279	0.645
IL-10	6.09(4.89–7.58)	5.2 (2.16–22.88)	4.45(2.31–6.48)	0.393	0.033
APRIL	152(56.68–194)	147(48.19–973)	74.19(35.96–175)	0.767	0.134
BAFF	157.5(139–266)	189.5(68.03–1011)	266(174–314)	0.491	0.055
CXCL13	27.23(14.07–57.17)	29.67(11.48–507)	12.52(10.43–33.96)	0.818	0.055
CCL2	736.5(656–771)	788(293–1868)	852(447–2665)	0.491	0.286
CXCL1	52(−63.15)	63.15(23.42–170)	34.92(−71.33)	0.155	0.668
IFN-α	2.73(1.94–5.86)	3.19(1.7–8.29)	2.72(1.94–5.86)	0.576	0.914
IL-1α	43.7(32.68–45.56)	68.91(39.47–215)	39.47(34.04–50.27)	0.026	0.522
IL-1β	1.42(0.71–1.83)	1.42(0.71–2.96)	1.24(1.05–1.42)	0.567	0.489
IL-12	124.5(− 133)	101(− 307)	56.35(−133)	0.766	0.831
VEGF-A	36.65(26.66–45.66)	52.68(32.13–119)	42.65(16.95–52.68)	0.026	0.67

*P* Paraneoplastic anti-NMDARE, *N* Anti-NMDARE without teratoma, *C* Negative control, *CCL* C-C motif chemokine ligand, *CXCL* C-X-C motif chemokine ligand, *IFN* interferon, *IL* interleukin, *TNF* tumour necrosis factor, *VEGF* vascular endothelial growth factor A, *APRIL* a proliferation-inducing ligand, *BAFF* B-cell-activating factor belonging to the tumour necrosis factor family, *G-CSF* granulocyte colony-stimulating factor, *GM-CSF* granulocyte-macrophage colony-stimulating factor

^a^Mann-Whitney U test and Bonferroni correction (α = 0.025)

## Discussion

Ovarian teratomas are the most common tumors in patients with paraneoplastic anti-NMDARE. The penetrance is reported to be 94% and mainly affects young women [19]. Our hospital-based demographic data showed lower occurrence rate of women (56%) and teratomas (7.5%) than reported in other regions (91, and 58%, respectively) [8]. This may be due to our different ethnic and gender ratio, however, patients with paraneoplastic anti-NMDARE presented a more severe illness than anti-NMDARE patients without tumors. Our data demonstrated a higher percentage of mechanical ventilation, intensive care, higher CSF antibody titres, and the late response to immune-therapy. Our data also showed that complete tumor resection and sufficient immune-therapy are important for successful outcome. This is consistent with a previous study showing a significantly better outcome in patients with early resection of teratomas compared to late or no resection [19].

Previous studies showed that tumors, especially teratomas, in patients with anti-NMDARE contained nervous tissues reacting with patients’ antibodies [9] and contained larger amounts of inflammatory infiltrates compared to teratomas from patients which did not develop anti-NMDARE [20]. These findings suggested that the teratomas were directly related to the pathogenesis of anti-NMDAR encephalitis. However, neural tissues are common in any teratoma, also in patients which do not present with symptoms of anti-NMDARE [4]. We carefully compared, therefore, the histopathology findings in teratomas obtained from different patient groups and found a peculiar population of dysmorphic neurons present in teratomas of patients with anti-NMDARE (10/11) but rarely of patients without anti-NMDARE (1/35). These dysmorphic neurons appeared irregular in their cell shape and had giant nuclei. They clustered in aggregates or scattered diffusely throughout the tumour tissue. A similar pathology finding was reported by Gregory et al. who showed that gangliogliomas, ganglioneuroblastoma, and neuronal abnormalities synonymous for the herein described population of dysmorphic neurons were absent from the control group [10]. In our control group, all cases had normal (mature) neurons, with the exception of one case also showing aggregates of dysmorphic neurons.

Our results showed that 90% of encephalitis-related teratomas were immunoreactive for antibodies directed against NR2A and NR2B epitopes. Specifically, the dysmorphic neurons in these teratomas had strong immunoreactivity for NMDAR subunits NR1, NR2A, and NR2B, and subsequent immunofluorescence showed consistent co-localisation of NMDAR subunits with IgG. In contrast, the neurons in most control cases showed negative or weakly positive for NMDAR subunits and no IgG-NMDAR co-localization, supporting the notion that aberrant IgG-NMDAR subunit expression in teratomas play an important role in the pathogenesis of anti-NMDARE [2, 3, 15, 18].

In the mixed germ cell tumours of case 12, normal neurons were replaced by a high density of cells with an atypical neuronal shape that stained positively for NeuN. Co-localization of IgG-NR2B was also detected. The results indicated that these cells may be the origin of NMDAR antibodies that lead to encephalitis. Though the tumor was resected, the patient quickly presented pulmonary metastasis and subsequently died of multiple organ failure. This case is very rare but demonstrates that even in malignant mixed tumours, neural cells expressing NMDA receptors are likely a key factor underlying the pathogenesis of anti-NMDARE.

Previous studies showed lymphocytes infiltrating into neural tissue of teratomas [5, 13, 20]. Though the adjacent localization of both cell components was not found in this study, inflammatory infiltrates were observed more frequently in cases with anti-NMDARE than controls, and CD20 labelled B-cells were predominant, further suggesting a humoral immune environment in the teratomas. Indeed, Mateusz et al. described that infiltrating lymphocytes in anti-NMDARE associated ovarian teratoma tissue could produce NR1-IgG in cell culture [15].

Although there was no statistical difference, it showed increasing trends of TNF-α, IL-10 and GM-CSF in patients with paraneoplastic anti-NMDARE than the negative symptom control, which indicated the involvement of T cell in pathogenesis of paraneoplastic anti-NMDARE. The result is similar to Kothur’s study [14]. Especially, the role of GM-CSF in autoimmune and inflammatory diseases gained rising attention recently. Increased levels of GM-CSF not only upregulate further production of proinflammatory cytokines from macrophages or dendritic cells, it also forms positive feedback, activates microglial cells, produces highly neurotoxic substances [16]. Moreover, TNF-α have a moderating effect on blood brain barrier (BBB) permeability via the internalization of tight junction proteins on endothelial cells [23] and GM-CSF is required for recruitment of peripheral myeloid cells that contribute to blood-brain and blood-spinal cord barriers disruption [1], which increases permeability and facilitates the transfer of serum antibodies through the BBB to the CSF. VEGF-A and IL-1α were higher in CSF of patients with anti-NMDARE but no tumor compared to those with paraneoplastic anti-NMDARE may suggest a different inflammation process.

## Contributions

We observed a lower occurrence rate of anti-NMDARE in Chinese patients with teratomas compared to other regions. However, patients with paraneoplastic anti-NMDARE presented clinically with a more serious illness than patients with anti-NMDARE and no tumours. We identified a peculiar cell population of dysmorphic neurons in teratomas with co-expression of NR1/NR2A/NR2B and IgG, and these neurons were candidate targets for autoimmune antibodies. Our current results will require further investigations, however, to experimentally confirm the role of the dysmorphic neurons with a floating-frog like appearance in paraneoplastic anti-NMDARE and the role of activating inflammatory cytokines.

## Supplementary information

**Additional file 1: Table S1-3.** Showed primary antibodies used in immunohistochemistry and immunofluorescence in the experiment; surgery finding, inflammatory features and NMDAR subunits expression in teratomas from patients without anti-NMDARE.

## Data Availability

The datasets used and/or analysed during the current study available from the corresponding author on reasonable request.

## Abbreviations

anti-NMDAR E: Anti–N-methyl-D-aspartate receptor encephalitis
NR: NMDA receptor
IgG: Immunoglobulin G
CASP2: Contactin-associated protein-like 2
LGI1: Leucine-rich glioma inactivated 1
GABAB-R: Gamma aminobutyric acid beta receptor
GAD65: Glutamic acid decarboxylase
AMPA1/2-R: Alpha-amino-3-hydroxy-5-methyl-4-isoxazole- propionic acid 1/2 receptor
DPPX: Dipeptidyl-peptidase-like protein-6
mGLURs: Metabotropic glutamate receptors
VGKC: Voltage gated potassium channel
VGCC: Avoltage gated calcium channel
mRS: Modified Rankin Scale
HE: Hematoxylin-Eosin
NeuN: Neuronal Nuclear epitope
MAP 2: Microtubule Associated Protein 2
GFAP: Glial Fibrillary Acidic Protein
FITC: Fluorescein isothiocyanate
TMRITC: Trimethyl rhodamine isothiocyanate
CCL: C-C motif chemokine ligand
CXCL: C-X-C motif chemokine ligand
IFN: Interferon
IL: Interleukin
TNF: Tumour necrosis factor
VEGF: Vascular endothelial growth factor A
APRIL: A proliferation-inducing ligand
BAFF: B-cell-activating factor belonging to the tumour necrosis factor family
G-CSF: Granulocyte colony-stimulating factor
GM-CSF: Granulocyte-macrophage colony-stimulating factor
SPSS: Statistic Package for Social Science
FLAIR: Fluid-attenuated inversion recovery
MTP: Methylprednisolone
PLAP: Placental alkaline phosphatase
AFP: Alpha fetal protein
HCG: Human chorionic gonadotropin
HPL: Human placental lactogen
CK: Creatine kinase
EMA: Epithelial membrane antigen
BBB: Blood brain barrier
