# Supplementary material for: Co-expression of NMDA-receptor subunits NR1, NR2A, and NR2B in dysplastic neurons of teratomas in patients with paraneoplastic NMDA-receptor-encephalitis: a retrospective clinico-pathology study of 159 patients
Source: Acta Neuropathol Commun. 2020 Aug 8;8:130. doi: 10.1186/s40478-020-00999-2 (PMC7414584; doi:10.1186/s40478-020-00999-2)
Supplement: Supplementary file 1 — Additional file 1: Table S1-3. Showed primary antibodies used in immunohistochemistry and immunofluorescence in the experiment; surgery finding, inflammatory features and NMDAR subunits expression in teratomas from patients without anti-NMDARE. [file 40478_2020_999_MOESM1_ESM.docx]

Supplemental table 1. Primary antibodies used in immunostaining and cell types stained

| Antibody | Source | Catalog No. | Dilution | Specific Cell Types, Stained |
| --- | --- | --- | --- | --- |
| NeuN | Abcam | ab104225 | 1:400 | Neurons |
| MAP-2 | Abcam | ab5392 | 1:200 | Neurons (dendrites), oligodendrocytes |
| GFAP | Abcam | ab53554 | 1:200 | Astrocytes |
| S 100 | Abcam | ab52642 | 1:200 | Neuroglia |
| NR1 | Alomone | AGC-001 | 1:300 | NMDA receptor 1 |
| NR2A | Alomone | AGC-002 | 1:200 | NMDA receptor 2A |
| NR2B | Alomone | AGC-003 | 1:200 | NMDA receptor 2B |
| Human IgG | SANTA | sc-69786 | 1:100 | All human IgG antibodies |
| CD3 | Abcam | ab16669 | 1:200 | T lymphocytes |
| CD20 | Invitrogen | 14-0202-82 | 1:200 | B lymphocytes |
| CD138 | MXB | MAB-0200 | 1:100 | Plasma cells |
| CD4 | ZSGB-BIO | ZA-0519 | 1:100 | Helper T lymphocytes |
| CD8 | ZSGB-BIO | ZA-0508 | 1:100 | Cytotoxic T lymphocytes |

NeuN, Neuronal Nuclear epitope; MAP2, Microtubule Associated Protein 2; GFAP, Glial Fibrillary Acidic Protein; NR, NMDA receptor; IgG, immunoglobulin G

Supplementary table 2. Surgery finding and inflammatory features of teratomas in patients without anti-NMDARE

| Group | age | sex | Pathology Findings | location | IgG | CD20 | CD138 | CD3 | CD4 | CD8 |
| --- | --- | --- | --- | --- | --- | --- | --- | --- | --- | --- |
| C1 | 24 | f | 4 × 4 ×2.2 cm Cystic mature teratoma | Left ovary | ++ | + | - | + | ++ | - |
| C2 | 36 | f | Diameter 20cm Immature teratoma (WHO Ⅲ) | Right ovary | +++ | - | - | + | + | + |
| C3 | 19 | f | 5 × 3.5 × 2 cm Cystic mature teratoma | Left ovary | ++ | - | - | + | ++ | + |
| C4 | 27 | f | 5.2 × 4 ×1.2cm Cystic mature teratoma | Right ovary | + | - | - | + | + | - |
| C5 | 27 | f | 3 × 2 × 1.7 cm Cystic mature teratoma | Left ovary | ++ | +++ | + | + | ++ | + |
| C6 | 17 | f | 7.5 × 6.5 × 3.5cm Cystic mature teratoma | Right ovary | +++ | + | - | - | ++ | + |
| C7 | 29 | f | 2 × 1.8 × 1 cm Cystic mature teratoma | Left ovary | +++ | ++ | - | ++ | ++ | - |
| C8 | 32 | f | 12 × 12 ×7cm Immature teratoma (WHO Ⅲ) | Left ovary | +++ | + | + | ++ | +++ | ++ |
| C9 | 36 | f | 4 × 3 ×1 cm Cystic mature teratoma | Left ovary | ++ | + | - | ++ | ++ | + |
| C10 | 23 | f | 6.5 × 3.8 × 2 cm Cystic mature teratoma | Left ovary | + | + | - | + | ++ | - |
| C11 | 20 | f | 4.5 × 2.8 × 2 cm Cystic mature teratoma | Left ovary | +++ | - | + | + | - | - |
| C12 | 18 | f | 6.2 × 5 × 2.8cm Cystic mature teratoma | Right ovary | ++ | - | - | + | ++ | ++ |
| C13 | 1 | f | 7× 5 × 3 cm Cystic mature teratoma | Left ovary | - | - | - | + | ++ | ++ |
| C14 | 7 | f | 6.2× 5.5 × 5.1 cm Cystic mature teratoma | Right ovary | +++ | + | - | + | ++ | + |
| C15 | 29 | f | 4.6× 2.5 × 2.2 cm Cystic mature teratoma | Right ovary | + | - | - | + | ++ | - |
| C16 | 21 | f | 12× 3.5 × 2.5 cm Cystic mature teratoma | Left ovary | + | - | - | + | +++ | +++ |
| C17 | 28 | f | 2.3 x 2 x 1.5 cm Cystic mature teratoma | Left ovary | + | - | - | + | + | - |
| C18 | 27 | f | 7× 6.3 × 1 cm Cystic mature teratoma | Left ovary | ++ | ++ | - | +++ | ++ | - |
| C19 | 42 | f | 5.5× 3.8 × 1.5 cm Cystic mature teratoma | Right ovary | +++ | ++ | + | + | ++ | - |
| C20 | 3 | f | 4.4× 2.9 × 2.6 cm Cystic mature teratoma | Right ovary | + | - | - | + | +++ | ++ |
| C21 | 25 | f | 8.6× 5 × 2.8 cm Cystic mature teratoma | Left ovary | - | - | - | - | ++ | - |
| C22 | 19 | f | 16.5× 13 × 8.5 cm Cystic mature teratoma | Right ovary | + | - | - | + | - | + |
| C23 | 29 | f | 7.5× 6.8 × 2.3 cm Cystic mature teratoma | Left ovary | + | - | - | + | ++ | + |
| C24 | 22 | f | 5.5× 5 × 4.5 cm Cystic mature teratoma | Right ovary | ++ | - | - | + | ++ | ++ |
| C25 | 24 | f | 4.3× 4.2 × 2.4 cm Cystic mature teratoma | Left ovary | + | - | - | - | - | - |
| C26 | 26 | f | 10.2× 8.5 × 5.3 cm Cystic mature teratoma | Right ovary | +++ | ++ | ++ | + | +++ | ++ |
| C27 | 41 | f | 6.5× 5 × 1.5 cm Cystic mature teratoma | Right ovary | +++ | ++ | - | + | ++ | - |
| C28 | 33 | f | 5.9× 5.9 × 3.5 cm Cystic mature teratoma | Right ovary | ++ | + | - | - | - | - |
| C29 | 22 | f | 11.5× 7.5 × 4.5 cm Cystic mature teratoma | Right ovary | ++ | - | - | - | - | - |
| C30 | 38 | f | 8× 6.2 × 2.5 cm Cystic mature teratoma | Left ovary | - | - | - | + | +++ | - |
| C31 | 20 | f | 8.3× 7.2 × 2.9 cm Cystic mature teratoma | Right ovary | ++ | ++ | - | + | +++ | ++ |
| C32 | 26 | f | 11× 10.8 × 5.4 cm Cystic mature teratoma | Right ovary | +++ | ++ | - | + | ++ | ++ |
| C33 | 35 | f | 5× 3.5 × 2 cm Cystic mature teratoma | Left ovary | +++ | - | + | + | +++ | ++ |
| C34 | 21 | f | 6.8× 5 × 4.8 cm Cystic mature teratoma | Right ovary | - | - | - | - | + | - |
| C35 | 27 | f | 4.5× 3.1 × 2.5 cm Cystic mature teratoma | Left ovary | + | ++ | - | ++ | +++ | ++ |

C, control, patients without anti-NMDARE; f, female; m, male; IgG, immunoglobulin; Cellular infiltrates: -, negative or positive cells less than 1% of microscopic field; +, <25%; ++, 25–50%; +++, 50–75%; ++++, >75%. positive IgG deposit: -, absent; +, mild; ++, moderate; +++ intense.

Supplementary table 3. NMDAR subunit analysis in neurons of teratomas without anti-NMDARE

| Case | Neuron staining | Neuron morphology | NRI | NR2A | NR2B |
| --- | --- | --- | --- | --- | --- |
| C1 | NeuN+ | Normal neurons | - | - | - |
| C2 | NeuN+ | Normal neurons | - | - | - |
| C3 | NeuN+ | Normal neurons | - | - | - |
| C4 | S100+ | Normal neurons | - | - | - |
| C5 | NeuN+ | Normal neurons | - | + | - |
| C6 | NeuN+ | Normal neurons | - | + | - |
| C7 | NeuN+ | Normal neurons | + | - | - |
| C8 | NeuN+ | Normal neurons | + | + | - |
| C9 | NeuN+ | Normal neurons | + | - | + |
| C10 | NeuN+ | Normal neurons | - | ++ | ++ |
| C11 | NeuN+ | Normal neurons | ++ | ++ | ++ |
| C12 | NeuN+ | clusters of dysmorphic neurons | - | - | - |
| C13 | MAP2+ S100+ | Normal neurons | + | + | - |
| C14 | NeuN+ | Normal neurons | + | - | ++ |
| C15 | NeuN+ | Normal neurons | - | - | - |
| C16 | NeuN+ | Normal neurons | + | ++ | - |
| C17 | NeuN+ | Normal neurons | - | + | - |
| C18 | MAP2+ S100+ | Normal neurons | - | - | - |
| C19 | MAP2+ | Normal neurons | - | - | - |
| C20 | MAP2+ | Normal neurons | - | - | - |
| C21 | MAP2+ S100+ | Normal neurons | - | - | - |
| C22 | MAP2+ | Normal neurons | + | ++ | + |
| C23 | MAP2+ | Normal neurons | - | + | - |
| C24 | MAP2+ | Normal neurons | - | ++ | + |
| C25 | NeuN+ | Normal neurons | + | + | - |
| C26 | NeuN+ | Normal neurons | - | + | + |
| C27 | MAP2+ S100+ | Normal neurons | - | - | - |
| C28 | NeuN+ | Normal neurons | - | + | ++ |
| C29 | NeuN+ | Normal neurons | - | + | + |
| C30 | MAP2+ | Normal neurons | - | - | - |
| C31 | NeuN+ | Normal neurons | - | - | - |
| C32 | MAP2+ | Normal neurons | - | + | + |
| C33 | NeuN+ | Normal neurons | - | - | - |
| C34 | NeuN+ | Normal neurons | - | - | ++ |
| C35 | MAP2+ | Normal neurons | - | - | - |

C1-12: patients without anti-NMDARE; -, positive cells less than 1% of microscopic field; + <25%; ++, 25–50%; +++, 50–75%; ++++, >75%. NeuN, Neuronal Nuclear epitope; MAP2, Microtubule Associated Protein 2; GFAP, Glial Fibrillary Acidic Protein; NR, NMDA reception
